# *Angiostrongylus cantonensis* in a Red Ruffed Lemur at a Zoo, Louisiana, USA

**DOI:** 10.3201/eid2805.212287

**Published:** 2022-05

**Authors:** Jessica Rizor, Ryan A. Yanez, Tuddow Thaiwong, Matti Kiupel

**Affiliations:** Michigan State University Veterinary Diagnostic Laboratory, Lansing, Michigan, USA

**Keywords:** Angiostrongylus, Angiostrongylus cantonensis, parasites, zoonoses, lemur, nematode, aberrant host, North America, Louisiana, United States

## Abstract

A red ruffed lemur (*Varecia rubra*) from a zoo in Louisiana, USA, was euthanized for worsening paresis. Brain and spinal cord histology identified eosinophilic meningoencephalomyelitis with intralesional adult *Angiostrongylus* sp. nematodes. PCR and sequencing confirmed *A. cantonensis* infection, indicating this parasite constitutes an emerging zoonosis in the southeastern United States.

*Angiostrongylus cantonensis* is a parasitic metastrongyloid nematode that has a neurotropic larval stage and is endemic throughout Southeast Asia and the Pacific Islands. The rat (*Rattus* spp.) is the main definitive host and a variety of gastropods serve as intermediate hosts. In rats, infections cause no brain damage and only some pulmonary disease in severe infections. However, in aberrant hosts, including humans and nonhuman primates, larvae cause severe eosinophilic meningoencephalitis. Clinical signs are associated with migration of the larvae and the immune response to dead or dying nematodes (*1*).

In 1987, *A. cantonensis* nematodes were detected in rats in New Orleans, Louisiana, USA (*2*); in 1995, a human case of eosinophilic meningitis was reported in North America in a child from New Orleans (*3*). *A. cantonensis* nematodes have now become endemic in the southeastern United States, as evidenced by reports of infection in a child in Texas (*4*); a horse from Mississippi (*5*); captive Geoffroy’s tamarins (*Saguinus geoffroyi*) in Alabama (*6*); and several animals in Florida, including a white-handed gibbon (*Hylobates lar*), an orangutan (*Pongo pygmaeus*), a white-throated capuchin monkey (*Cebus capucinus*), a red ruffed lemur (*Varecia rubra*), and a nine-banded armadillo (*Dasypus novemcinctus*) (*7*,*8*). Ingestion of infected gastropods and paratenic hosts or unwashed contaminated vegetables are proposed routes of infection for aberrant hosts.

The International Union for Conservation of Nature lists red ruffed lemurs (*Varecia rubra*) as critically endangered (*9*). In June 2021, a 9-year-old male red ruffed lemur from a zoo in Louisiana was humanely euthanized because of hind limb paresis and a right head tilt that worsened over an 8-day period. The lemur was housed in a troop of 5 adult lemurs in an outdoor exhibit. Various snail species are common in the enclosure, but no other lemurs were clinically affected. 

A necropsy performed at the Michigan State University Veterinary Diagnostic Laboratory (Lansing, Michigan, USA) identified no gross lesions. The laboratory formalin-fixed and processed the brain, the entire spinal cord, and all major organs for histopathology. Histopathologic examination revealed multiple transverse and longitudinal sections of adult nematodes within the subarachnoid space and neuropil of the cerebellum and brainstem. Nematodes were ≈50–70 μm in diameter and had a 3–4-μm thick smooth, eosinophilic cuticle and prominent lateral cords. Adult nematodes had coelomyarian musculature, and the pseudocoelom contained a reproductive tract and an intestinal tract lined by multinucleated cells with flocculent eosinophilic to brown material in the lumen (Figure). Nematodes were surrounded by hemorrhage and small numbers of eosinophils, neutrophils, macrophages, and glial cells. Several cerebellar folia were effaced by invading nematodes, hemorrhage, and inflammation. The cerebellar meninges were expanded by numerous eosinophils, fewer neutrophils, foamy macrophages, multinucleated giant cells, and lymphocytes. A representative section of thoracic spinal cord contained an identical single adult nematode in the subdural space. Another adult nematode had regionally effaced the dorsal horn in a section of lumbar spinal cord. The affected spinal cord had regional rarefaction of both gray and white matter and marked variation in myelin sheath size. The spinal cord meninges were similarly expanded by moderate numbers of eosinophils, lymphocytes, plasma cells, and fewer eosinophils.

**Figure:**
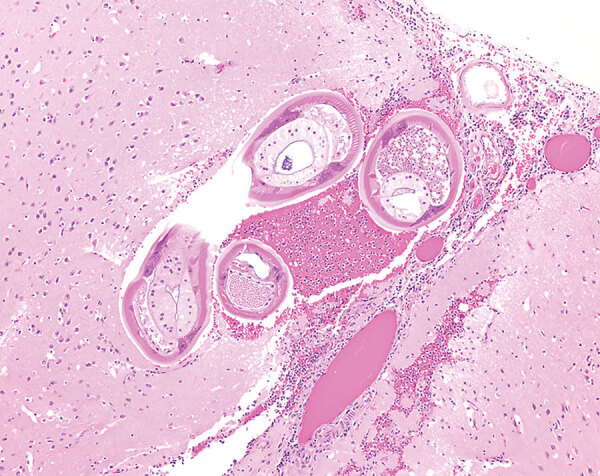
Formalin-fixed brainstem specimen from red ruffed lemur (*Varecia rubra*) infected with *Angiostrongylus cantonensis* nematodes at a zoo in Louisiana, USA. Hematoxylin and eosin stain shows adult nematodes measuring ≈50–70 μm in diameter with 3–4 μm thick, smooth, eosinophilic cuticle and prominent lateral cords. Nematodes have a coelomyarain musculature and a pseudocoelom that contains a reproductive tract and an intestinal tract, lined by multinucleated cells. Original magnification ×10.

We suspected *Angiostrongylus* sp. nematode infection on the basis of histomorphologic findings and anatomic features of migrating nematodes. We extracted nematode DNA by using a QIAamp DNA FFPE Tissue Kit (QIAGEN, https://www.qiagen.com) following the manufacturer’s instructions. We performed species identification by PCR on paraffin-embedded brain tissue using primers (forward 5′-TGA AAT CGT TGA AGT GGA ACC-3′ and reverse 5′-GTC GCA ACC TGT ACG CTC TAC-3′) that we designed specifically to amplify an ≈500-bp product of the 28S ribosomal RNA gene. Sanger sequencing of the amplicon revealed >99% similarity to *A. cantonensis* (GenBank accession no. AY292792.1), 92% to *A. vasorum* (GenBank accession no. AM039758.1), and 91% to *A. chabaudi* (GenBank accession no. KM216825.1).

In the southeastern United States, *A. cantonensis* nematodes have emerged as clinically significant parasites in mammals, including humans, causing severe neurologic disease and death. Our findings illustrate another example of a nonhuman primate succumbing to infection and should raise awareness of the potential risk for infection in endemic areas. Diagnosing *A. cantonensis* infection in a live patient is challenging because of nonspecific clinical signs, ineffective serologic testing, and inability to detect adult nematodes in cerebrospinal fluid. Real time PCR performed on cerebrospinal fluid has detected DNA remnants of larvae in 22 of 33 human patients with eosinophilic meningitis (*10*). Because diagnosing and treating *A. cantonensis* infection is difficult, awareness and prevention are key. Humans and animals should only consume thoroughly cleaned vegetables and fully cooked gastropods and paratenic hosts. Persons living in affected areas can reduce risks for invasive gastropod species to become established by protecting food storage areas and local gardens from rats and gastropods. 

In conclusion, the *A. cantonensis* nematode is emerging in the southeastern United States, and its range seems to be expanding. Because this parasite can infect a wide variety of mammals, including humans, both human and veterinary caretakers should remain vigilant for this zoonotic pathogen.
